# Floor Eggs in Australian Cage-Free Egg Production

**DOI:** 10.3390/ani15131967

**Published:** 2025-07-04

**Authors:** Ruby Putt, Hubert Brouwers, Peter John Groves, Wendy Isabelle Muir

**Affiliations:** 1 Poultry Research Foundation, The University of Sydney, Camden 2570, Australia; ruby.putt@sydney.edu.au (R.P.); hubert.brouwers@sydney.edu.au (H.B.); peter.groves@sydney.edu.au (P.J.G.); 2 School of Life and Environmental Sciences, The University of Sydney, Camperdown 2050, Australia; 3 Sydney School of Veterinary Science, The University of Sydney, Camden 2570, Australia

**Keywords:** cage-free, floor eggs, laying hen, egg production, free range, mislaid eggs, nest, ground eggs, ventilation, flock size

## Abstract

Cage-free egg production is now the predominant system in Australia. However, the occurrence of floor eggs (FE), which are eggs laid outside designated nest boxes, presents a major challenge for these producers. To understand factors that may be associated with the laying of FE, a national scoping survey of cage-free egg-laying flocks was undertaken. Forty-three flocks across multiple farms were surveyed via a phone-based interview using predetermined questions. Floor egg levels ranged from 0.01–17%. There was no difference in floor egg levels between the breeds of brown-egg-laying hens. Age at peak lay did not alter the level of FE, but higher rate of peak lay had a weak association with fewer FE (r = −0.31, *p* = 0.049). Larger flocks had a lower percentage of FE (r = −0.5, *p* = 0.002), and farmers of larger sized flocks considered a lower level of floor eggs to be acceptable. Farms with tunnel-ventilated sheds reported fewer FE compared to those using other ventilation systems (*p* = 0.013). Higher floor egg levels were associated with increased labor costs (*p* = 0.023). These findings suggest that shed design and environmental management may be leveraged to reduce floor egg occurrence and improve operational efficiency in cage-free systems.

## Simple Summary

Cage-free egg farming is becoming more common in Australia, now making up the majority of egg sales. These systems are seen as better for animal welfare, but they also bring new challenges for farmers. One issue is floor eggs, which are eggs laid by hens outside of the nest boxes. These eggs often cannot be collected easily or used for sale, leading to lost income and more work for staff. In this study, we looked at 43 flocks across Australia to find out what factors might lead to more floor eggs. We found that the number of floor eggs varied widely, from almost none to 17% of daily egg production. Larger flocks tended to have fewer floor eggs, and sheds with tunnel ventilation systems had lower levels of floor eggs compared to those using natural types of airflow. Farms with more floor eggs also had higher labor costs. These findings suggest that some features of the shed and how it is managed can influence the number of floor eggs. Understanding these factors can help farmers manage their systems, reduce waste, and produce eggs more efficiently while still meeting animal welfare expectations.

## 1. Introduction

The poultry industry plays a vital role in addressing global food security, with egg production being a key contributor to providing high-quality dietary protein. In Australia, egg production continues to grow, with 6.98 billion eggs produced in the 2023–2024 financial year, contributing to a total sales value of $1370.1 million AUD [1]. As consumer demand evolves, the Australian egg industry is expected not only to meet the rising demand for food but also to ensure that it is produced under systems that support animal welfare [2].

This shift in consumer expectations has led to significant changes in production methods. Cage-free systems have rapidly gained prominence, accounting for 78.3% of total retail egg sales by volume at the end of the 2023–2024 financial year. Within this category, free-range systems made up 57.4% and barn systems 20.9%, while cage systems represented 19.7%, down by 7% from the previous year [1]. While cage-free systems offer welfare benefits, including increased opportunity for hens to express natural behaviors such as dustbathing, preening, and foraging [3,4,5], they also pose new management challenges. Compared to conventional cage systems, cage-free hens experience greater freedom of movement, but this can result in increased energy demands [6] and lower production efficiency per unit of input [7].

One of the most pressing challenges in cage-free systems is the incidence of mislaid or floor eggs (FE), that is, eggs laid outside of designated nest boxes. These eggs are more prone to contamination, breakage, and labor-intensive collection [8] and can contribute to a loss of up to 10% in daily production if unmanaged [9,10]. Mislaid eggs represent a multifaceted problem, influenced by a combination of biological, environmental, and managerial factors. Genetic factors are likely contributors; however, findings on breed-related differences in the level of FE at peak lay remain mixed. While some studies have observed similar rates across typical brown-egg-laying breeds [11,12], others report significant differences between strains [13]. In Australia, where only brown egg strains are used commercially, the influence of genetics within this group remains underexplored.

In addition to genetics, the scale and management capacity of an operation may influence the level of FE at peak lay. Smaller farms may face resource limitations that affect their ability to monitor, adjust, and respond to system issues, potentially increasing the incidence of FE [14]. Larger enterprises may be better equipped to implement corrective strategies through investment in infrastructure and data systems [15].

The shed environment also plays a critical role in influencing laying behaviors. Poor ventilation and inadequate temperature control, particularly in hot climates, can induce heat stress, reducing nest use and overall productivity [16,17]. Stressed hens tend to avoid higher levels in multi-tiered systems, further increasing the likelihood of floor laying [18,19].

This study aimed to capture a snapshot of the current state of the level of FE at peak lay across commercial cage-free farms in Australia. A national phone-based survey was conducted to gather information on housing design, flock demographics, breed, environmental management, and the level of FE at peak lay. These findings serve as a foundational dataset to guide more detailed follow-up investigations into the underlying causes of mislaid eggs in cage-free systems.

## 2. Materials and Methods

### 2.1. Ethics Approval

This project was conducted at The University of Sydney, Camden Campus. All experimental procedures were approved by The University of Sydney Human Ethics Committee, protocol number 2023/150 and were in accordance with the National Health and Medical Research Council’s (NHMRC) National Statement on Ethical Conduct in Human Research (2018) and the NHMRC’s Australian Code for the Responsible Conduct of Research (2018).

### 2.2. Experimental Design

The secure browser-based Electronic Data Capture (EDC) software REDCap was used for the development, distribution, data collection, and management of the survey [20,21]. Survey questions were formatted and implemented into REDCap as the primary survey record, in addition to the ‘inform to consent’ form and ‘individual farm recording’ form. A call for participation in the study was shared with egg farmers by a third party, the funding body Australian Eggs. Famers could volunteer to be involved by scanning a QR code or clicking on a web link, which took them to the consent form. Only farmers who volunteered to be involved were included in the short phone survey for initial data capture, and they were able to withdraw from the survey at any stage.

For each participating flock on each farm, a broad but brief phone-based survey was conducted, which lasted for approximately 10–20 min. Data collection was completed through a conversational methodology using a predetermined questionnaire (see Table A1). Briefly, the contents of the questions were as follows: system type (i.e., free-range, cage-free), house ventilation type, hen breed, flock size, flock age, age at peak lay, rate of lay at peak lay, percentage of FE, acceptable level of FE at peak lay, and whether the level of FE increased labor costs. However, the phone-call-based methodology also allowed for additional ad lib responses. The data were separated by flock, i.e., each farm with multiple flocks completed a separate survey record for each flock on the farm. The collection of results was conducted by a dedicated surveyor, whereby questions were asked methodically as outlined in a predetermined format detailing the proposed questions and sequence. As each question was asked and the responses given, the surveyor entered the responses into REDCap as a new record, first assigning to each the relevant farm and flock identifier. After all the survey questions were discussed, the record was marked as completed and stored in the REDCap software awaiting analysis. Each survey record had to be marked and completed prior to conducting the next survey.

### 2.3. Data Storage and Access

All the data were directly lodged into the REDCap software and managed in accordance with responsible data management practices. The REDCap software is an approved program by The University of Sydney, supporting the collection of sensitive data in a secure encrypted manner [20]. Raw data access was only provided to the surveyors and data analysts, that is, individuals who required access for the purposes of the study, which was approved by the lead investigator. All the participants were given the option to view the study results following de-identification of farms and statistical analysis; however, they had no access to the raw data.

### 2.4. Statistical Analysis

All the survey responses were assorted and tabulated automatically within the REDCap ‘Data Export’ function. Columns contained the questions, and the responses were within the rows. Before analysis, the raw data were inspected for completeness and accuracy. One survey response was removed due to significant incompleteness in responses for ‘floor egg prevalence at peak lay’, ‘rate of lay’, and ‘age at peak lay’. Under the variable for ‘shed type’, the choices were split into the following groups for purposeful analysis: natural or tunnel ventilation, aviary, colony nest, deep litter; and cage-free, free-range, and pasture. In instances where a response was provided as a numerical range, the maximum number was taken for analysis in factors including ‘floor egg prevalence at peak lay’ and ‘acceptable floor egg prevalence’. All the categorical variables were standardized to maintain consistency across the datasets. All cleaning procedures were conducted using Excel. All the farms and flocks were de-identified prior to analysis. A one-way ANOVA was performed to compare means across multiple groups, while independent *t*-tests were used for pairwise comparisons. A factorial ANOVA was used to assess for any interaction between flock size and tunnel ventilation. Pearson’s correlation analysis was conducted to evaluate relationships between the variables, and the equation of the line was determined through linear regression. All statistical tests were carried out using GenStat (version 23.1; VSN International (VSNi)) with significance set at *p* < 0.05.

## 3. Results

### 3.1. Characteristics of the Participating Flocks

Data were collected from 43 flocks across Australia, including the states of New South Wales, Queensland, Tasmania, and Western Australia. Most of the respondents, 33 flocks, were in New South Wales, encompassing 77% of all the flocks included in the survey. The remaining 23% of flocks included five flocks from Queensland, two flocks from Tasmania, and three flocks from Western Australia. In some cases, large egg farms had multiple flocks. Across all flocks (*n* = 43), the level of FE at peak lay ranged between 0.01 and 17%, with an average of 3.45% and a median of 2.5%. The number of acceptable FE at peak lay nominated by the farmer for each flock ranged between 0.2 and 10%. The most common cage-free housing system of flocks entered in this survey was free range (*n* = 41), as opposed to pasture based (*n* = 2). In these systems, the reported level of FE at peak lay was 3.52% and 1.75%, respectively.

Age at peak lay ranged from 24 to 58 weeks of age (WOA), with an average of 31 WOA and a median of 28 WOA, based on data from 34 flocks. The statistical analysis revealed an extremely weak and statistically insignificant relationship between age at peak lay and the level of FE at peak lay (y = 0.004411x + 2.937; 0.95 confidence interval, r = 0.01, *p* = 0.94). Additionally, the rate of lay at peak lay varied amongst flocks between 67.97% and 98.3%, with an average of 88.6% and a median of 90%, based on 36 flocks. There was a weak negative correlation of rate of lay at peak lay with FE, that is, as rate of lay at peak lay increased, the level of FE at peak lay decreased (y = 11.52 − 0.09927x; 0.95 confidence interval, r = −0.31, *p* = 0.049).

### 3.2. Breed of Hen

The three main breeds of egg-laying hens used in Australia were captured in this study. These were Hy-Line Brown (*n* = 19 flocks), Lohmann Brown (*n* = 5 flocks), and ISA Brown (*n* = 19 flocks). There was no statistically significant difference between these breeds in the level of FE at peak lay (*p* > 0.05) (Table 1).

### 3.3. Flock Size

The size of flocks captured in this study ranged from 200 to 33,300 hens, with an average of 13,407 and median of 10,000 hens. For statistical analysis, the flocks were divided according to flock size into quartiles (Q). The range of flock sizes for each quartile, from smallest (Q1) to largest (Q4) are presented in Table 2. The average number of hens in each quartile was Q1 = 1740, Q2 = 8236, Q3 = 15,962 and Q4 = 29,000. The average FE at peak lay for each quartile was Q1 = 7.15%, Q2 = 3.39%, Q3 = 2.15% and Q4 = 1.26%, illustrating a negative correlation of FE with flock size (y = 5.9571 − 0.0002x; 0.95 confidence interval, r = −0.50, *p* = 0.002). That is, as flock size decreased, FE at peak lay increased. Additionally, with an ANOVA, the smaller flocks, i.e., Q1, had statistically higher levels of FE at peak lay compared to Q3 and Q4 (*p* < 0.05) (Table 2), whereas the level of FE for Q2 flocks was similar to those of all the other quartiles. Similarly, the level of FE at peak lay considered to be acceptable by the farmer had a negative correlation with flock size (y = 3.790 − 9.031e − 005x; 0.95 confidence interval, r = −0.42, *p* = 0.006). That is, as flock size decreased, the acceptable level of FE at peak lay increased. Of note, the acceptable level of FE at peak lay between Q1 and Q4 was approaching statistical significance (*p* = 0.055).

### 3.4. Ventilation

The type of shed ventilation impacted the level of FE. Specifically, flocks in sheds that were tunnel-ventilated (mechanical ventilation) had a significantly lower average level of FE at peak lay (1.73%) compared to sheds that were naturally ventilated (4.67%) (*p* = 0.013) (Table 3).

### 3.5. Flock Size and Ventilation

A factorial ANOVA was conducted to investigate whether there was an interaction between flock size and the presence of tunnel ventilation on the percentage of floor eggs. In this analysis, Q1 (Table 2) was excluded as tunnel ventilation was not reported with any of these flocks. Hence, the analysis involved flock sizes Q2, Q3, and Q4 with or without tunnel ventilation (Table 4). It should be noted that the mean values for floor eggs for both flock size quartiles and tunnel ventilation in Table 4 differ from those reported in Table 2 and Table 3. This discrepancy is due to the method used to calculate the mean within this analysis. For example, for each flock size quartile, the data were initially allocated into tunnel ventilation subgroups (‘Yes’ or ‘No’), and the floor egg percentage for that flock size quartile was then calculated from the mean of the two tunnel ventilation subgroups. This applied to the calculation of floor eggs for both the main treatments of flock size quartile and tunnel ventilation.

As presented in Table 4, both flock size (*p* = 0.02) and tunnel ventilation (*p* = 0.01) had significant independent effects on the percentage of floor eggs. Specifically, there was no interaction between flock sizes Q2, Q3, and Q4, and tunnel ventilation on the level of floor eggs (*p* = 0.76), indicating that their effects were additive rather than interactive.

Further, it should be noted that for the calculated level of floor eggs in Q2 and Q4 from this analysis (Table 4), Q2 had higher FE (3.81%) compared to Q4 (1.65%; *p* = 0.02). Floor egg levels in tunnel-ventilated sheds remained lower than in sheds that were not tunnel ventilated (1.56% compared to 3.35%, respectively; *p* = 0.01).

### 3.6. Labor Costs

For the nine flocks where an increase in labor costs due to FE was reported, the average level of FE was 5.95%, significantly higher than the 2.78% FE in flocks that did not experience a change in labor costs (*p* = 0.023) (Table 5). However, a change in labor costs was not impacted by flock size (*p* = 0.278).

### 3.7. Health Challenges

Throughout the course of conducting this survey, the discussion often led to respondents commenting on any health issues experienced by the flock. It is to be noted that respondents were not directly asked to identify disease events in the flock and hence these data cannot be used to develop causative conclusions, but this aspect should be more fully explored in a subsequent deep-dive survey. For 6 of the 43 flocks included in this survey, the farmer reported a health-related challenge that was either present or had been experienced within their flock during the laying period. The average level of FE in these flocks was 7.67%, compared to 2.76% for all other flocks.

## 4. Discussion

This study investigated the prevalence and contributing factors of floor eggs in Australian cage-free egg production systems using data collected from 43 commercial farms via a structured phone survey. Key variables examined included breed of hen, flock size, ventilation type, housing system design, labor costs, and health challenges. The aim was to identify management and environmental factors associated with higher floor egg incidence, informing some management approaches that could contribute to reducing FE and improving overall productivity in cage-free systems.

### 4.1. Role of Hen Breed on Floor Eggs

Of the flocks included in this study the level of FE at peak lay was not influenced by the breed of brown-egg-laying hens, which also represent those most often used in Australia: Hy-Line Brown, Lohmann Brown, and ISA Brown (*p* > 0.05; Table 1). This suggests that breed is not a determining factor for FE during peak lay. In contrast to this finding, a recent study, although not originally designed to investigate the effect of breeds on FE, observed a difference in FE between brown-egg-laying breeds. In that case, ISA Brown flocks were more likely to produce ≥2% FE compared to the Hy-Line and Lohmann Brown breeds [13]. However, as the authors mention, this observation may have been confounded by sampling locality and the study time frame [13]. Another study observed almost identical, non-significant differences in number of FE between Hy-Line Brown and Bovans Brown hens [11]. They also observed this pattern between two white-egg-producing hen breeds (DeKalb White and Hy-Line W36) [11].

Contrastingly, genetic differences associated with varying laying behavior are more commonly seen when comparing brown- and white-eggshell-producing hen breeds, rather than within brown-egg-laying strains [12]. During peak lay periods, brown-egg-laying strains had higher odds of non-nest laying (1.56) compared to white-egg-laying strains (0.76) [11]. Additionally, a difference in the percentage of eggs laid outside nest areas has been observed between Spanish brown- (Red-barred Vasca & Red Villafranquina) and white-egg-producing breed (Black Castellana) [12]. However, in this case, the Spanish brown-egg-laying strain exhibited a reduced prevalence of mislaid eggs (8.24% and 11.55% respectively) compared to the white-egg-laying strain (19.79%) [12]. Thus, when taken together, recent findings indicate inconsistency in the impact of breed of egg-laying hen on the laying of FE.

### 4.2. Impact of Flock Size on Floor Eggs

Due to the intensive nature of the egg production industry and driven by the increased demand for global consumption of eggs, their success is becoming increasingly reliant on adaptability, research interest and implementation, and the uptake of technological processes [22]. Within this study, a strong negative correlation was found between flock size and level of FE (r = −0.50, *p* = 0.002). Similarly, acceptable floor egg levels were also negatively correlated with flock size (r = −0.42, *p* = 0.006). These findings suggest that larger flocks may benefit from more standardized management, improved infrastructure, or greater access to technological resources, which may contribute to reducing FE laying. Smaller-scale egg production may introduce financial limitations for the exploration of technological developments in farm- and flock-based monitoring [23]. This is especially relevant for the collection, storage, and maintenance of flock data. The lack of technological uptake, for example precision farming, by smaller-scale farms is largely due to the cost of its implementation and the farms comparatively smaller disposable income for these investments, compared to larger operations [24,25]. Furthermore, the absence of these technologies may introduce limitations to their production potential with regards to animal welfare, real-time monitoring, and overall farm sustainability [26,27]. Although not evaluated in this study, the disparity between farm- and flock-based monitoring could explain the differences in reported floor egg values between flocks of different sizes. At the very least, this relationship warrants further exploration. Given the practical challenges faced by small and medium-sized cage-free egg producers, especially regarding labor and infrastructure limitations, the identification of simple, cost-effective interventions is critical. Nest box type and individual hen preference can influence both laying behaviors and the location of egg deposition [28,29]. Hens tend to be relatively consistent in their laying site preference throughout their productive life, whether they favor nest boxes or floor areas [30]. Therefore, strategies that restrict access to alternative laying areas, such as the floor, combined with efforts to enhance nest box attractiveness are key to reducing floor laying and encouraging proper nest use with the aim of reducing floor egg levels.

Several strategies have been proposed that may help reduce floor egg prevalence without requiring significant investment. These include the use of a substrate inside the nest boxes to cater to hen preference for comfort and overall attractiveness, and installing curtains or visual barriers to provide hens with additional privacy and perceived safety while laying [31,32]. Additionally, frequent collection of FE and blocking habitual laying spots may prevent floor-laying behaviors from becoming established patterns [31,33]. While these interventions appear promising, further controlled research is necessary to evaluate their effectiveness and feasibility across diverse farm sizes and housing systems. The suitability of such low-cost interventions may be particularly relevant for smaller operations that cannot justify investment in large-scale automated systems. However, it is not known whether the small to medium-sized flocks included in this study were already implementing some of these interventions, or whether such strategies contributed to the observed levels of FE. This uncertainty highlights the need for future studies to document greater detail of specific management practices across flock sizes.

### 4.3. Effect of Ventilation on Floor Eggs

In this study the effects of tunnel compared to natural ventilation types on FE were explored. Tunnel ventilation facilitates high-rate longitudinal airflow through the shed, driven by exhaust fans positioned at one end and air inlets or evaporative cooling pads at the opposite end. This system is designed to enhance thermal comfort and air quality, particularly during periods of heat stress, which may influence bird behavior and laying patterns. Observations from this study identified that compared to natural ventilation, housing having tunnel ventilation may reduce the level of FE within the flock, i.e., the type of shed ventilation impacted the level of FE (4.67% compared to 1.73%, respectively; *p* = 0.013, Table 3, and 3.35% compared to 1.56%, respectively; *p* = 0.01, Table 4). Flocks housed in sheds using natural ventilation, for example curtain sides, may have reduced egg production performance when compared to flocks in tunnel-ventilated housing [34,35,36]. Tunnel ventilation can better regulate and maintain a lower temperature during hotter ambient climates compared to natural ventilation types [35,36,37]. Additionally, tunnel ventilation has been widely used as an effective management tool in preventing and controlling heat stress and production loss amongst flocks. Within these systems, the forced airflow allows for convective heat loss from the surface of the bird’s body [38]. Without effective ventilation, heat stress has detrimental consequences on a bird’s productive efficiency, health, and welfare [18,35]. Under conditions of heat stress, birds will prioritize biological functioning to assist thermoregulation with attempts to reduce their core body temperature [17,34]. Under these conditions, hens spend less time walking and are less likely to traverse enrichment within their environment (i.e., perches and ramps) whilst spending more time drinking and resting [18]. As a result, inadequate ventilation and the resulting heat stress not only impair hen activity but may also contribute to an increased likelihood of FE, as hens alter their laying behaviors to dissipate heat. Although no current studies directly link floor egg prevalence with heat stress or ventilation design, the evidence presented highlights the need for further research to explore these relationships, given that heat stress is known to affect other laying behaviors [39].

### 4.4. Housing System

Of the surveyed flocks, the majority were housed in free-range systems (*n* = 41), with very few in mixed-agriculture, pasture-based (*n* = 2) systems. The average level of FE at peak lay in the free-range flocks was 3.52%, while in the pasture-based systems it was 1.75%. However, due to the large disparity in sample sizes, statistical comparisons between the systems could not be evaluated. Notably, the two pasture-based flocks were among the smallest surveyed, each consisting of only 200 hens. Given that the Australian cage-free egg industry is predominantly made up of commercial free-range operations [1], these findings likely reflect the broader industry structure, in which mixed-agriculture systems are comparatively fewer and smaller in scale. Additionally, both free-range and pasture-based systems enhance welfare through increased behavioral freedom [40,41]; they both may also create additional laying sites, particularly outdoors, where mislaid eggs can be lost, contaminated, or damaged [42,43]. Further, some mislaid eggs may not be readily identified amongst the pastures in those systems. Consequently, the level of FE reported for these systems may be an underestimation. Our findings underscore the need for research that quantifies floor egg losses associated with outdoor access and investigates management strategies to mitigate these challenges.

### 4.5. Labor Costs Associated with Floor Eggs

Flocks that experienced increased labor costs due to FE (*n* = 9) had significantly higher floor egg levels at peak lay (5.95%) compared to those with no change to labor costs (2.78%) (*p* = 0.023; Table 5). Interestingly, there was no significant difference in flock size between these two groups (*p* = 0.278). Any increase in the cost of running the business is a major concern for all animal production systems [44]. The management of additional costs on the overall farming operation may be influenced by the accuracy and consistency of the financial data collected, as well as the methods used to collect and analyze this information. Throughout the surveys, the smaller systems having smaller flocks generally appeared to have less reliable systems for recording data, such that the answers to some questions were less decisive and it appeared to be more difficult to provide a definite value for the impact of FE on their business. In these cases, many responses were based on estimates rather than quantitative data. In contrast, many respondents of the larger flock sizes were more critical of their finances and had data collected in real time, locating balance sheets and loss calculations, providing a more informed answer to this question. Although, in this study, flock size did not influence the effect of FE on overall labor costs, the small sample size and absence of multiple linear regression analysis means we cannot rule out an effect of flock size on the additional costs of labor with FE.

### 4.6. Impact of Health Challenges on Floor Eggs

As previously mentioned, throughout the course of each survey the discussion often led to respondents commenting on any health issues experienced by the flock, which should be more fully explored in a subsequent deep-dive survey. Additionally, there is a risk that cannibalism may be under-reported, since this figure is based on farmer observation and is thus prone to underestimating prevalence where the problem occurs at a low level [45]. Six flocks (14%) reported recent or ongoing health issues—primarily cannibalism and spotty liver disease (SLD). These flocks had a markedly higher average level of FE at peak lay (7.67%) compared to all the others (2.76%), although the sample size limits statistical testing. Health challenges like cannibalism and SLD are known to impair productivity and welfare [46,47,48]. While causality cannot be confirmed, the notable difference in the level of FE at peak lay between healthy and health-compromised flocks signals a possible link worth exploring in more targeted investigations.

### 4.7. Study Limitations

This research was conducted using a phone survey, with responses recorded by the researcher, which introduces several inherent limitations. First, the reliance on self-reported data may be subject to recall bias and social desirability bias, potentially affecting the accuracy of the respondents’ answers [49,50]. The response rate was modest, with 43 responses, and limited to those within the reach of Australian Eggs, which may influence the relevance of the findings to the wider target population of flocks held in cage-free housing systems. Moreover, while the survey instrument was carefully developed, there remains the possibility of misinterpretation of questions by the respondents or ambiguity in responses [51]. Finally, external factors at the time of data collection (such as seasonal influences or specific socio-economic conditions) might have impacted the responses, further limiting the applicability of the results to different contexts or periods [52].

Some management and environmental variables that may influence floor egg prevalence were not captured in this survey, including lighting provision, nest box size and placement, nest-to-hen ratio, and stocking density. Additionally, regional climate differences across participating farms may have influenced ventilation effectiveness and hen behavior but were not explicitly accounted for in the analysis. Similarly, information on the source of the flock and rearing practices was not collected. Whether birds were reared from day-old chicks or acquired as pullets, and the nature of their rearing environment, may shape laying site preferences and early behavioral development and hence floor egg prevalence [31]. Future research should aim to include a broader set of flock and farm-level variables, particularly those related to rearing conditions, lighting, nest availability, and stocking density. These gaps will be addressed in a more detailed follow-up survey currently in development.

The findings of this study provide novel insights into the multifactorial nature of FE in Australian cage-free systems. They highlight factors at farm level (flock size, ventilation, and labor cost) and possibly bird level (health) that impact floor egg production, many of which may be amenable to management intervention. Several areas for further research have also been identified, which together would inform targeted mitigation strategies in the management of FE.

## 5. Conclusions

This study highlights the variable incidence of FE in cage-free egg production systems, ranging from 0.01% to 17% of daily egg production. No significant differences in the level of FE at peak lay were observed between the three brown-egg-producing breeds studied—Hy-Line Brown, ISA Brown, and Lohmann Brown—suggesting that breed alone may not be a major contributing factor. Flock size and ventilation type were found to independently affect the level of floor eggs. Interestingly, larger flocks (20,001–33,300 hens) and those housed in tunnel-ventilated sheds exhibited the lowest level of FE, indicating that both scale and environmental control may play protective roles. Not surprisingly, increased labor costs were associated with higher FE, though this may be confounded by other factors including flock size. Finally, health events, such as cannibalism and spotty liver disease, may increase the risk of floor egg laying, warranting further targeted research to better understand their potential impact.

## Figures and Tables

**Table 1:** Floor eggs at peak lay for brown-egg-laying hens in Australia.

Breed	Floor Eggs (%)	SEM	*n*	*p*-Value
Hy-Line Brown	4.00	1.02	19	
Lohmann Brown	4.09	3.26	5	
ISA Brown	2.73	0.35	19	0.56

ANOVA tested using *p* < 0.05 as the confidence threshold. SEM = standard error of mean. *n* = number of flocks.

**Table 2:** Percentage of floor eggs and acceptable percentage of floor eggs at peak lay based on flock size quartile.

Flock Size, Presented in Quartile Grouping	Floor Eggs (%)	SEM	Acceptable Floor Eggs at Peak Lay (%)	SEM	*n*
Q1, 200–3000 hens	7.15 ^a^	1.82	3.4 *	1.09	10
Q2, 3001–9999 hens	3.39 ^a,b^	0.35	3.3	0.53	11
Q3, 10,000–20,000 hens	2.15 ^b^	0.51	2.4	0.43	13
Q4, 20,001–33,300 hens	1.26 ^b^	0.41	1 *	0	9
*p*-Value	0.002		0.055		

^a,b^ rows with different superscripts are different at *p* < 0.05. SEM = standard error of mean. *n* = number of flocks. * Indicated values approaching statistical significance.

**Table 3:** Floor eggs in flocks housed in sheds with or without tunnel ventilation.

Tunnel Ventilation	Floor Eggs (%)	SEM	*n*	*p*-Value
No *	4.67 ^a^	0.92	25	
Yes	1.73 ^b^	0.35	18	0.013

* Natural ventilation. ^a,b^ rows with different superscripts are different at *p* < 0.05. SEM = standard error of mean. *n* = number of flocks.

**Table 4:** Factorial ANOVA of flock size and tunnel ventilation on percentage of floor eggs.

Treatment	Floor Eggs (%)	SEM	*n*
Flock Size			
Q2	3.81 ^a^	0.55	11
Q3	1.92 ^a,b^	0.49	13
Q4	1.65 ^b^	0.57	9
Tunnel Ventilation			
No	3.35 ^A^	0.48	15
Yes	1.56 ^B^	0.40	18
Interaction			
Q2 × No	4.71	0.93	3
Q2 × Yes	2.90	0.57	8
Q3 × No	2.53	0.54	9
Q3 × Yes	1.30	0.81	4
Q4 × No	2.82	0.93	3
Q4 × Yes	0.48	0.66	6
*p*-Value			
Flock Size	0.02		
Tunnel Ventilation	0.01		
Flock Size × Tunnel Ventilation	0.76		

^a,b^ rows with different superscripts are different at *p* < 0.05. ^A,B^ rows with different superscripts are different at *p* < 0.05. SEM = standard error of mean. *n* = number of flocks.

**Table 5:** The level of floor eggs in flocks where floor eggs increased the cost of labor.

Increase in Labor Costs Due to Floor Eggs	Floor Eggs (%)	SEM	*n*	*p*-Value
No	2.78 ^a^	0.46	34	
Yes	5.95 ^b^	2.13	9	0.023

^a,b^ rows with different superscripts are different at *p* < 0.05. SEM = standard error of mean. *n* = number of flocks.

## Data Availability

The original contributions in this study are included in the article; further inquiries can be directed to the corresponding author.

## Abbreviations

The following abbreviations are used in this manuscript:

FE	floor eggs
SLD	spotty liver disease
WOA	weeks of age

## Appendix A

**Table A1:** Tabulated output of ‘primary survey record’ for questions posed. Table details the survey questions and response options.

Prompt/Questions	Response Options	Response Category
Record ID	[⋯⋯⋯]	Open Response
Flock number	[⋯⋯⋯]	Open Response
Flock ID as a combination of farm ID and flock number	[⋯⋯⋯]	Open Response
Is this flock housed at the same address as before? (from the consent form)	Yes No	Yes/No
Location of shed if not at home farm	[⋯⋯⋯]	Open Response
What is the shed number	[⋯⋯⋯]	Open Response
What is the type of shed in which the flock is housed? e.g., Free range, natural ventilation, free range tunnel ventilation, aviary or other	Natural Ventilation Tunnel ventilation Aviary Colony nest Deep litter Cage-free Free-range Pasture Other	Tick boxes
Expand if shed type is ‘other’ or notes are required.	[⋯⋯⋯]	Open Response
What is the breed of layer hen?	[⋯⋯⋯]	Open Response
How many hens are in this flock	[⋯⋯⋯]	Open Response
What is the current age of the flock?	[⋯⋯⋯]	Open Response
At what age did the flock reach peak lay?	[⋯⋯⋯]	Open Response
What was the rate of lay at peak lay? (format in %)	[⋯⋯⋯]	Open Response
What was the level of floor eggs at peak lay?	[⋯⋯⋯]	Open Response
Did the level of floor eggs in this flock cause notable increases in labor costs?	Yes No	Yes/No
What level of floor eggs are acceptable at peak lay?	[⋯⋯⋯]	Open Response
